# Ethnic variation in cancer patients’ ratings of information provision, communication and overall care

**DOI:** 10.1080/13557858.2015.1126561

**Published:** 2016-02-07

**Authors:** Lorna Trenchard, Louise Mc Grath-Lone, Helen Ward

**Affiliations:** ^a^ School of Public Health, Imperial College London, London, UK; ^b^ Patient Experience Research Centre, School of Public Health, Imperial College London, London, UK

**Keywords:** Cancer, patient experience, ethnicity, communication, information provision

## Abstract

**Objective**. Ethnic inequalities in cancer patient experience exist but variation within broad ethnic categories is under-explored. This study aimed to describe variation by ethnic sub-category in experiences of information provision and communication (key domains of patient experience) using National Cancer Patient Experience Survey (NCPES) data.

**Design**. The NCPES 2012–2013 contained responses from 68,737 cancer patients treated at 155 NHS Trusts in England. Multivariate logistic regression was used to investigate associations between ethnicity and patients’ ratings of overall care, information provision and communication.

**Results**. Variation by and within broad ethnic categories was evident. Non-White patients (particularly Asian patients (OR_adj_:0.78; 95%CI:0.67-0.90, *p*=0.001)) were less likely than White patients to receive an understandable explanation of treatment side effects. Among Asian patients, those of Bangladeshi ethnicity were least likely to receive an understandable explanation.

**Conclusions**. Effective communication and information provision are important to ensure patients are well informed, receive the best possible care and have a positive patient experience. However, ethnic inequalities exist in cancer patients’ experiences of information provision and communication with variation evident both between and within broad ethnic categories. Further work to understand the causes of this variation is required to address ethnic inequalities at practice and policy level.

## Introduction

Cancer care and treatment is a resource-intensive area of healthcare which is set to expand, due in part to an ageing population. It is also becoming more complex as treatment options increase and more patients have co-morbidities. As a result, many countries, including the UK (where healthcare is available free of charge to ordinary residents through the National Health Service (NHS)), have developed and implemented strategies designed to ensure the best possible cancer care and treatment is available to all patients (New Zealand Ministry of Health 2002; Department of Health 2010; National Cancer Equality Initiative 2010; Koh, Graham, and Glied 2011; Department of Health 2011a). Despite these strategies, patients’ experience of cancer care is known to vary by socio-demographic factors such as ethnicity (Ayanian et al. 2005; Shadmi et al. 2010; Penner et al. 2013). Ethnic disparities in patient experience are well documented in the NHS, with many studies finding that non-White patients (especially Chinese and Asian groups) report less positive experiences than White British groups across a range of healthcare services (Raleigh et al. 2007; Sizmur 2011; Lyratzopoulos et al. 2012; Henderson, Gao, and Redshaw 2013), including cancer care (Quality Health 2013; Bone et al. 2014).

In order to facilitate health service improvements, the Department of Health has identified a number of areas that are important to patient experience, a key domain of high-quality healthcare (Department of Health 2011b). Information provision has been identified as being critical to good patient experience (NHS National Quality Board 2012) leading to a focus on high-quality written and verbal communication with the aim of effectively informing and empowering patients and their families. However, different ethnic groups are known to have different preferences for information provision and communication; for example, there is variation between ethnic groups in the preferred amount of information and mode of delivery (e.g. printed, online and person-to-person) (Thomas et al. 1999; Kakai et al. 2003; Mitchison et al. 2012). Ethnic minority patients who do not speak the native language of the healthcare setting also report communication issues (Ayanian et al. 2005; Shadmi et al. 2010). We therefore hypothesised that there would be wide variation in reported patient experience related to communication and information provision among cancer patients in England.

The National Cancer Patient Experience Survey (NCPES) is undertaken in England annually on behalf of the Department of Health to evaluate NHS cancer services and contains several questions related to patients’ experiences of communication and information provision. While published NCPES reports have identified inequalities in reported patient experience by ethnicity in areas such as overall ratings of and access to care (Department of Health 2009), there have not been any detailed analyses of ethnic variation in communication. Furthermore, each broad ethnic category used for analysis in these reports represents a patient population containing several diverse groups (i.e. the Asian ethnic group contains Indian, Pakistani and Bangladeshi patients), yet inequalities are not explored by small ethnic sub-category. The aim of this study was to gain further insight into variation in cancer patients’ reported experiences of information provision and communication, through analysis of relevant NCPES data by ethnic categories and sub-categories.

## Methods

### Sources of data

Secondary analysis was performed on data from the 2012–2013 NCPES, conducted by Quality Health on behalf of the Department of Health. The 2012–2013 NCPES was sent to every adult (aged over 16 years) with a primary diagnosis of cancer, admitted as an inpatient or day case patient for treatment in an NHS hospital in England and discharged between 1 September and 30 November 2012. Questionnaires were not sent to patients who were known to have died since discharge. Non-responders were followed up with two postal reminders (Quality Health 2012). The 2012–2013 NCPES data set contained responses to 70 multiple-choice questions and associated demographic information for 68,737 cancer patients from 155 English NHS Trusts, the administrative bodies responsible for commissioning primary, community and secondary health services from providers.

### Patient, clinical and Trust-level factors

Patient, clinical and Trust-level characteristics are known to impact on ratings of patient experience (Bone et al. 2014) and were adjusted for in our analyses. Self-reported data was used for these factors where possible as it is considered to be ‘gold-standard’, particularly for ethnicity (Saunders et al. 2013). From NCPES responses, self-reported patient (i.e. gender, age, ethnicity, presence of a long-standing condition, sexual orientation and employment status) and clinical characteristics (i.e. time since diagnosis and response to treatment) were extracted. Hospital-reported data was used for NHS Trust, tumour group, patient classification (day case or inpatient) and index of multiple deprivation (IMD), a composite measure of area-level deprivation associated with a patient's postcode (Department for Communities and Local Government 2011). Analysis of the data by ethnicity was performed using six broad categories and fourteen sub-categories (Table 1). Chinese and Other were included as separate broad categories in line with other studies showing different rates of patient satisfaction in these groups (Department of Health 2009; Sizmur 2011; El Turabi et al. 2013). White and White British were used as reference categories for the broad and sub-categories, respectively, as they contained the largest number of participants.

**Table 1.** Selected characteristics of respondents to 2012–2013 NCPES.

	*n*	%	Tumour group	*n*	%
Gender					
Male	31,060	45.2	Brain/central nervous system	730	1.1
Female	35,358	51.4	Breast	13,916	20.2
			Colorectal/lower gastrointestinal	8899	12.9
Age group			Gynaecological	3896	5.7
16–25	257	0.4	Haematological	11,602	16.9
26–35	904	1.3	Head and neck	2437	3.5
36–50	6150	8.9	Lung	5018	7.3
51–65	20,324	29.6	Other	2739	4.0
66–75	22,539	32.8	Prostate	5585	8.1
76+	15,571	22.7	Sarcoma	720	1.0
			Skin	1854	2.7
Ethnicity^a^			Upper gastrointestinal	4283	6.2
White	63,434	92.3	Urological	7058	10.3
* White British*	*61,260*	*89.1*			
* White Irish*	*936*	*1.4*	Time since first treatment		
* Any other White background*	*1238*	*1.8*	<1 year	43,687	63.6
			1–5 years	16,498	24.0
Mixed	323	0.5	>5 years	5647	8.2
* White and Black Caribbean*	*90*	*0.1*			
* White and Black African*	*40*	*0.1*	Employment status		
* White and Asian*	*107*	*0.2*	Full time employment	10,861	15.8
* Any other Mixed background*	*86*	*0.1*	Part time employment	5868	8.5
			Homemaker	1805	2.6
Asian or Asian British	1185	1.7	Student	178	0.3
* Indian*	*624*	*0.9*	Retired	41,339	60.1
* Pakistani*	*250*	*0.4*	Unemployed, seeking work	466	0.7
* Bangladeshi*	*61*	*0.1*	Unable to work for health reasons	3773	5.5
* Any other Asian background*	*250*	*0.4*	Other	1424	2.1
					
Black or Black British	923	1.3	Patient classification		
* Caribbean*	*515*	*0.7*	Day case	44,295	64.4
* African*	*364*	*0.5*	Inpatient	24,442	35.6
* Any other Black background*	*44*	*0.1*			
			Cancer response to treatment		
Chinese	170	0.2	Has responded fully (no signs/symptoms)	24,442	35.5
			Has been treated but is still present	17,089	24.5
Any other ethnic group	112	0.2	Has not been treated at all	1166	1.7
			Has come back after it was treated	3564	5.2
Long-standing conditions^b^			Has responded fully, but have a new cancer	1926	2.8
None	40,403	58.8	Not certain what is happening	13,996	20.4
Deafness or hearing impairment	7040	10.2			
Blindness or visually impairment	1533	2.3	National IMD Quintile		
Physical condition	9045	13.2	1 (Least deprived)	16,413	23.9
Learning disability	290	0.4	2	16,412	23.9
Mental health condition	1339	2.0	3	14,492	21.1
Long-standing illness^c^	8917	13.0	4	11,840	17.2
			5 (Most deprived)	9221	13.4

^a^Ethnic sub-categories are italicised.

^b^Patients may have multiple long-standing conditions (therefore the column total may exceed 100%).

^c^Such as (but not limited to) HIV, diabetes or chronic heart disease. Gender was unknown for 3.4% of respondents (*n* = 2319), age group for 4.4% (*n* = 2992), ethnicity for 3.8% (*n* = 2590), long-standing conditions for 9.0% (*n* = 6175), time since first treatment for 4.2% (*n* = 2905), employment status for 4.4% (*n* = 3023), response to treatment for 9.6% (*n* = 6574) and IMD quintile for 0.5% (*n* = 359).

### Data analysis

The 2012–2013 NCPES was screened for questions related to overall care, information provision and communication. For the 23 questions selected (Appendix), patients’ responses were coded into binary outcomes (‘positive’ or ‘not positive’) as per the survey guidance (Quality Health 2013). Firstly, univariate logistic regression was used to explore associations between a positive rating of overall care (as assessed by Q70 ‘Overall how would you rate your care?’) and patient and clinical factors, including ethnicity. A mixed effects multivariate model, adjusting for confounders, was then developed by including fixed effects for patient and clinical factors associated with a positive overall care rating at a significance level of *p* < .05 and a random effect for NHS Trust of treatment. This multi-level regression framework accounts for differences that may arise because ethnic minority patients are clustered in poorly performing Trusts. Finally, a likelihood ratio test was used to compare models using the six broad ethnic categories and fourteen sub-categories and to determine if variation within broad ethnic groups was statistically significant, taking into account the number of respondents. The same method was used to explore the associations between patients’ ratings of overall care and being given the right amount of information (as measured by Q67 ‘How much information were you given about your condition and treatment?’).

The remaining 21 questions were then grouped by the stage of the pathway to which they referred (i.e. diagnosis, diagnostic tests, treatment side effects, operations, support or leaving hospital) as indicated by the section of NCPES questionnaire in which they appeared (Appendix). Composite scores by broad and sub-categories of ethnicity were determined for each stage of the pathway by calculating an unweighted average of the proportion of positive responses to the constituent questions. While this method does not address variation in the meaning of a positive response to a question or the relative contribution of a question to overall patient experience, it is consistent with the NCPES survey guidance and provides an overview of variation in reported patient experience. Based on this exploration of the data, information related to treatment side effects was selected for further analysis as it was the lowest rated part of the cancer care pathway. Univariate and multivariate multi-level logistic regression was performed as previously described to examine associations between ethnicity and the three NCPES questions related to information about treatment side effects (Q17, 18 and 19) adjusting for potential confounding factors.

Communication with staff is an important domain of patient experience and ratings of communication vary by staff group (Quality Health 2013); however, ethnic differences have not been described. Therefore, three comparable questions which asked how often patients received understandable answers to important questions from different staff members (Q24, 37 and 41) were also selected for further analysis. Associations with ethnicity were explored as previously described using univariate and multivariate multi-level logistic regression. All statistical analyses were performed using Stata v12 and complete case analysis was undertaken (i.e. respondents with missing demographic, clinical or Trust-level data, or those who did not answer the question of interest, were excluded).

## Results

### Respondent characteristics and rating of care

In total, 68,737 patients completed 2012–2013 NCPES representing a response rate of 64% (Quality Health 2013). Most respondents were female, aged 66–75 years and described themselves as White British (Table 1).

### Overall rating of care

A large proportion of respondents (88%, *n* = 58,525) rated their overall care positively (i.e. as ‘very good' or ‘excellent’). However, there was variation between ethnic categories with non-White patients (particularly Chinese and Asian patients) significantly less likely to rate their care positively compared to White patients (Table 2). There was also variation in overall care rating within broad ethnic categories by sub-category. For example, among Asian patients Bangladeshis had the lowest rating of overall care (54.2% vs.72.4% of Indian patients, *p* = .01) and among White patients those of ‘Any other background' rated their care less positively than British patients (82.4% vs. 89.6%, *p* = .001). These differences by ethnicity in reported patient experience persisted after adjusting for potential confounders such as age, gender and NHS Trust of treatment.

**Table 2.** Proportion of patients rating their overall care positively, by ethnic broad and sub-category.

Ethnicity	*n*	%	OR	95% CI	*p*-Value	OR_adj_^a^	95% CI	*p*-Value
*Broad ethnic category*								
White (all)	52,664	89.4	(ref)^b^			(ref)^b^		
Mixed (all)	258	81.0	0.50	0.37–0.69	**<.001**	0.53	0.38–0.73	**<.001**
Asian or Asian British (all)	955	73.6	0.33	0.29–0.38	**<.001**	0.34	0.29–0.40	**<.001**
Black or Black British (all)	669	78.9	0.44	0.37–0.53	**<.001**	0.48	0.40–0.59	**<.001**
Chinese	140	72.9	0.31	0.21–0.47	**<.001**	0.33	0.22–0.48	**<.001**
Any other ethnic group	92	75.0	0.35	0.22–0.57	**<.001**	0.43	0.26–0.71	**.001**
*Ethnic sub-category*								
White British	50,901	89.6	(ref)^c^			(ref)^c^		
White Irish	741	88.0	0.85	0.68–1.06	.16	0.88	0.70–1.11	.28
Any other White background	1022	82.4	0.55	0.46–0.64	**<.001**	0.58	0.49–0.69	**<.001**
White & Black Caribbean	74	81.1	0.50	0.37–0.69	**<.001**	0.49	0.27–0.89	**.02**
White & Black African	30	76.7	0.38	0.28–0.89	**.02**	0.41	0.17–0.99	**.05**
White & Asian	85	82.4	0.54	0.31–0.95	**.03**	0.55	0.31–0.98	**.05**
Any other Mixed background	69	81.2	0.50	0.27–0.91	**.02**	0.51	0.28–0.95	**.03**
Indian	497	72.4	0.33	0.25–0.37	**<.001**	0.31	0.25–0.38	**<.001**
Pakistani	199	73.4	0.33	0.24–0.44	**<.001**	0.31	0.22–0.43	**<.001**
Bangladeshi	48	54.2	0.15	0.08–0.24	**<.001**	0.16	0.09–0.29	**<.001**
Any other Asian background	211	81.0	0.51	0.35–0.70	**<.001**	0.55	0.38–0.78	**.001**
Caribbean	368	77.2	0.41	0.31–0.50	**<.001**	0.43	0.33–0.55	**<.001**
African	270	81.5	0.52	0.38–0.70	**<.001**	0.53	0.38–0.73	**<.001**
Any other Black background	31	77.4	0.41	0.17–0.96	**.04**	0.47	0.20–1.11	.09
Chinese	140	72.9	0.31	0.21–0.47	**<.001**	0.33	0.22–0.48	**<.001**
Any other ethnic group	92	75.0	0.35	0.22–0.57	**<.001**	0.43	0.26–0.71	**.001**

Note: Analysis was restricted to 54,778 respondents and excluded patients who did not respond to Q70 (*n* = 2538) or had missing data for age, gender, ethnicity, time since first treatment, long-standing condition status, response to treatment or IMD quantile (*n* = 11,421). Significant associations (*p* < .05) are highlighted in bold. According to the likelihood ratio test, the model using ethnic sub-categories was significantly better than the model using broad categories (*χ* ^2^(10) = 52.00, *p* < .001).

^a^Adjusted for age, gender, long-standing condition, time since first treatment, tumour group, patient classification, response to treatment, IMD and Trust.

^b^For comparisons by broad ethnic categories, the reference group was White patients.

^c^For comparisons by ethnic sub-categories, the reference group was White British patients.

### Communication and information provision

Patients who felt they received the right amount of information (i.e. responded positively to Q67) were far more likely to rate their overall care as ‘very good' or ‘excellent' (OR_adj_:7.74, 95%CI:7.25–8.25, *p* < .001), adjusting for age, gender, ethnicity, long-standing condition, time since first treatment, tumour group, response to treatment, patient classification (day case or inpatient) and Trust. However, patients’ ratings of communication and information provision varied across the stages of the cancer care pathway, as well as by ethnicity (Table 3). Overall, patients reported being most satisfied with information provision when leaving hospital and least satisfied with information about the side effects of treatments. Chinese and Asian patients (particularly Bangladeshi patients) were less satisfied with communication and information provision across the entire pathway.

**Table 3.** Composite positive scores for questions related to communication and information provision along the cancer care pathway by ethnic broad and sub-category.

	Diagnosis (%)	Diagnostic tests (%)	Treatment side effects (%)	Operations (%)	Support (%)	Leaving hospital (%)
All patients	72.0	84.2	70.6	71.5	79.4	88.9
*Broad ethnic category*						
White (all)	72.3	84.6	70.8	71.6	79.5	88.9
Mixed	67.4	79.7	67.4	68.0	78.4	86.7
Asian or Asian British	64.4	74.8	68.2	68.4	75.0	89.0
Black or Black British	65.6	77.1	65.5	70.1	79.0	86.6
Chinese	57.8	72.3	67.0	64.4	67.1	86.5
Any other ethnic group	67.1	74.3	70.2	67.9	73.8	82.8
*Ethnic sub-category*						
White British	72.3	84.6	70.7	71.5	79.5	89.0
White Irish	73.4	87.0	74.4	75.2	82.2	89.1
Any other White background	73.3	82.7	72.9	73.5	78.3	87.2
White & Black Caribbean	67.8	81.6	66.7	69.8	75.0	89.4
White & Black African	68.8	88.2	70.3	64.8	86.1	90.7
White & Asian	63.9	75.0	66.3	67.1	72.8	84.7
Any other Mixed background	70.5	79.7	68.3	68.6	83.6	84.4
Indian	64.7	72.5	67.1	68.5	73.5	89.3
Pakistani	63.1	76.9	67.8	67.2	75.5	86.1
Bangladeshi	55.3	64.2	55.3	61.6	62.8	78.5
Any other Asian background	67.4	80.7	74.4	70.8	80.4	93.9
Caribbean	66.4	76.6	64.7	67.2	77.9	85.8
African	64.7	77.5	65.7	73.5	79.6	86.7
Any other Black background	63.9	79.6	72.8	71.9	86.1	95.7
Chinese	57.8	72.3	67.0	64.4	67.1	86.5
Any other ethnic group	67.1	74.3	70.2	67.9	73.8	82.8

Note: Composite positive scores were calculated as the unweighted average of the proportion of positive respondents for the constituent questions (as described in Appendix).

### Information about side effects of treatment

Three NCPES questions related to information about the side effects of treatment asked patients if they had been (a) given an understandable explanation of potential side effects (Q17), (b) given written information that was easy to understand about the side effects of their treatment (Q18) and (c) told about potential side effects that may develop in the future (Q19). Variation in responses to these questions by ethnicity was evident (Table 4). Black and Asian (especially Bangladeshi) patients were less likely than White patients to report that they had received an explanation of side effects that they could understand from staff or been told about side effects that might develop in the future. A similar pattern was seen with regards to receiving understandable written information about the side effects of treatment. Among patients who reported that they were not given written information about the side effects of treatment that was ‘easy to understand’, 27.1% (*n* = 2436) reported that they had been given written information but found it difficult to understand. Adjusting for age, gender, long-standing condition, tumour group, time since first treatment, response to treatment, patient status, IMD and Trust, non-White participants were less likely to report that they found the written information provided easy to understand (e.g. Black patients OR_adj_:0.36, 95%CI:0.28–0.47, *p* < .001).

**Table 4.** Variation in communication about the side effects of treatment, by ethnicity.

Ethnicity	*n*	%	OR	95% CI	*p*-Value	OR_adj_^a^	95% CI	*p*-Value
(a) Given an understandable explanation of side effects of treatment^b^								
*Broad ethnic category*								
White	37,649	75.3	(ref)^c^			(ref)^c^		
Mixed	176	72.1	0.85	0.64–1.12	.25	0.83	0.62–1.10	.20
Asian or Asian British	658	70.5	0.78	0.68–0.90	**.001**	0.78	0.67–0.90	**.001**
Black or Black British	454	69.5	0.75	0.63–0.89	**.001**	0.74	0.63–0.89	**.001**
Chinese	93	69.9	0.76	0.53–1.10	.15	0.72	0.50–1.06	.09
Any other ethnic group	68	75.6	1.01	0.63–1.64	.96	1.04	0.64–1.70	.88
*Ethnic sub-category*								
White British	36,304	75.2	(ref)^d^			(ref)^d^		
White Irish	571	79.3	1.26	1.05–1.51	**.01**	1.30	1.08–1.56	**.01**
Any other White background	774	78.0	1.16	1.00–1.36	**.05**	1.16	0.99–1.35	.06
White & Black Caribbean	49	70.0	0.77	0.46–1.28	.31	0.76	0.46–1.28	.31
White & Black African	24	80.0	1.32	0.54–3.22	.55	1.26	0.51–3.13	.61
White & Asian	62	76.5	1.08	0.64–1.80	.78	1.06	0.63–1.79	.82
Any other Mixed background	41	65.1	0.61	0.37–1.03	.07	0.61	0.36–1.03	.06
Indian	337	69.3	0.75	0.61–0.91	**.003**	0.75	0.61–0.91	**.01**
Pakistani	143	73.7	0.92	0.67–1.27	.63	0.89	0.65–1.24	.50
Bangladeshi	21	45.7	0.28	0.16–0.50	**<.001**	0.30	0.17–0.55	**<.001**
Any other Asian background	157	75.9	1.03	0.75–1.42	.84	1.05	0.76–1.45	.79
Caribbean	249	70.0	0.75	0.60–0.94	**.01**	0.77	0.61–0.98	**.03**
African	181	69.1	0.74	0.57–0.96	**.02**	0.71	0.54–0.93	**.01**
Any other Black background	24	72.7	0.88	0.41–1.89	.74	0.88	0.40–1.91	.75
Chinese	93	69.9	0.76	0.53–1.10	.15	0.72	0.50–1.06	.09
Any other ethnic group	68	75.6	1.01	0.63–1.64	.96	1.04	0.64–1.70	.88
(b) Given understandable written information about side effects of treatment^e^								
*Broad ethnic category*								
White	40,568	82.6	(ref)^c^			(ref)^c^		
Mixed	195	80.6	0.87	0.64–1.20	.41	0.71	0.51–0.99	**.04**
Asian or Asian British	705	77.6	0.73	0.62–0.85	**<.001**	0.64	0.54–0.75	**<.001**
Black or Black British	494	78.5	0.77	0.64–0.94	**.01**	0.66	0.54–0.81	**<.001**
Chinese	102	76.1	0.67	0.45–1.00	.05	0.51	0.34–0.78	**.002**
Any other ethnic group	73	82.0	0.96	0.56–1.65	.86	0.73	0.42–1.28	.27
*Ethnic sub-category*								
White British	39,159	82.3	(ref)^d^			(ref)^d^		
White Irish	573	82.5	0.99	0.82–1.21	.95	1.06	0.86–1.30	.58
Any other White background	836	85.5	1.25	1.04–1.49	**.02**	1.09	0.90–1.31	.38
White & Black Caribbean	52	78.8	0.79	0.44–1.42	.42	0.68	0.37–1.26	.25
White & Black African	23	79.3	0.81	0.33–1.99	.65	0.64	0.25–1.64	.35
White & Asian	67	81.7	0.94	0.54–1.65	.84	0.72	0.40–1.28	.26
Any other Mixed background	53	81.5	0.93	0.50–1.75	.83	0.78	0.41–1.49	.45
Indian	365	76.8	0.70	0.57–0.87	**.001**	0.63	0.50–0.78	**<.001**
Pakistani	140	75.7	0.66	0.47–0.92	**.02**	0.58	0.41–0.83	**.002**
Bangladeshi	30	66.7	0.42	0.23–0.79	**.01**	0.43	0.22–0.82	**.01**
Any other Asian background	170	83.3	1.06	0.73–1.53	.77	0.84	0.57–1.22	.36
Caribbean	268	78.4	0.77	0.60–0.99	**.04**	0.73	0.55–0.95	**.02**
African	199	78.0	0.75	0.56–1.01	.06	0.57	0.42–0.78	**<.001**
Any other Black background	27	84.4	1.14	0.44–2.97	.79	0.89	0.34–2.36	.82
Chinese	102	76.1	0.67	0.45–1.00	.05	0.51	0.34–0.78	**.002**
Any other ethnic group	73	82.0	0.96	0.56–1.65	.86	0.73	0.42–1.28	.27
(c) Told about side effects that may develop in the future^f^								
*Broad ethnic category*								
White	25,505	55.4	(ref)^c^			(ref)^c^		
Mixed	122	51.9	0.87	0.67–1.13	.29	0.83	0.64–1.8	.16
Asian or Asian British	502	56.6	1.05	0.92–1.20	.47	1.03	0.90–1.19	.66
Black or Black British	314	50.9	0.84	0.71–0.98	**.03**	0.80	0.68–0.95	**.01**
Chinese	61	45.9	0.68	0.49–0.96	**.03**	0.64	0.45–0.90	**.01**
Any other ethnic group	49	56.3	1.04	0.68–1.59	.84	1.10	0.71–1.70	.66
*Ethnic sub-category*								
White British	24,574	55.2	(ref)^d^			(ref)^d^		
White Irish	392	60.0	1.22	1.04–1.43	**.02**	1.23	1.05–1.45	**.01**
Any other White background	539	58.3	1.14	0.99–1.30	.06	1.14	1.00–1.31	.05
White & Black Caribbean	36	57.1	1.08	0.66–1.78	.76	1.02	0.61–1.70	.93
White & Black African	16	55.2	1.00	0.48–2.07	.99	0.94	0.45–1.98	.88
White & Asian	36	44.4	0.65	0.42–1.01	.05	0.62	0.40–0.97	**.04**
Any other Mixed background	34	54.8	0.98	0.60–1.62	.95	0.96	0.58–1.59	.87
Indian	240	52.8	0.91	0.75–1.09	.29	0.89	0.74–1.08	.25
Pakistani	113	59.8	1.21	0.90–1.61	.21	1.13	0.84–1.52	.43
Bangladeshi	18	40.0	0.54	0.30–0.98	**.04**	0.58	0.31–1.06	.08
Any other Asian background	131	66.2	1.59	1.18–2.13	**.002**	1.61	1.19–2.17	**.002**
Caribbean	164	48.7	0.77	0.62–0.95	**.02**	0.76	0.61–0.95	**.02**
African	133	53.6	0.94	0.73–1.20	.61	0.87	0.67–1.13	.30
Any other Black background	17	53.1	0..92	0.46–1.84	.81	0.93	0.46–1.89	.85
Chinese	61	45.9	0.68	0.49–0.96	**.03**	0.64	0.45–0.90	**.01**
Any other ethnic group	49	56.3	1.04	0.68–1.59	.84	1.10	0.71–1.70	.66

Note: Significant associations (*p* < .05) are highlighted in bold.

^a^Adjusted for age, gender, long-standing condition, time since first treatment, tumour group, patient classification, response to treatment, IMD quintile, Trust location (in/outside Greater London) and Trust.

^b^Analysis was restricted to 52,029 respondents and excluded patients who did not respond to Q17 (*n* = 5031) or had missing data for age, gender, ethnicity, time since first treatment, long-standing condition status, response to treatment or IMD quintile (*n* = 11,677). According to the likelihood ratio test, the model using ethnic sub-categories was significantly better (at 95% confidence level) than the model using broad categories (*χ* ^2^(10) = 28.77, *p* = .001).

^c^For comparisons by broad ethnic categories, the reference group was White patients.

^d^For comparisons by ethnic sub-categories, the reference group was White British patients.

^e^Analysis was restricted to 51,115 respondents and excluded patients who did not respond to Q18 (*n* = 6413) or had missing data for age, gender, ethnicity, time since first treatment, long-standing condition status, response to treatment or IMD quintile (*n* = 11,209). According to the likelihood ratio test, the model using ethnic sub-categories was not significantly better (at 95% confidence level) than the model using broad categories (*χ* ^2^(10) = 6.75, *p* = .75).

^f^Analysis was restricted to 48,025 respondents and excluded patients who did not respond to Q19 (*n* = 10,230) or had missing data for age, gender, ethnicity, time since first treatment, long-standing condition status, response to treatment or IMD quintile (*n* = 10,482). According to the likelihood ratio test, the model using ethnic sub-categories was significantly better (at 95% confidence level) than the model using broad categories (*χ* ^2^(10) = 28.51, *p* = .002).

### Understandable answers to important questions

Three NCPES questions asked patients how often they received understandable answers to important questions from different staff members, namely (a) Clinical Nurse Specialists (CNSs) (Q24), (b) doctors (Q37) and (c) ward nurses (Q41). Overall patients reported that CNSs were most likely to give responses to questions that were easy to understand and, for all three staff groups, worse communication was reported by non-White patients (Table 5). These differences by ethnicity remained after adjusting for potential confounders, such as age, tumour group and Trust, and again Bangladeshi patients had particularly low ratings.

**Table 5.** Receiving understandable answers to important questions from staff, by ethnicity.

Ethnicity	*n*	%	OR	95% CI	*p*-Value	OR_adj_^a^	95% CI	*p*-Value
(a) Received an understandable answer from a clinical nurse specialist^b^								
*Broad ethnic category*								
White	35,953	91.2	(ref)^c^			(ref)^c^		
Mixed	173	86.1	0.57	0.38–0.85	**0.01**	0.67	0.44–1.00	.05
Asian or Asian British	586	83.2	0.46	0.38–0.56	**<.001**	0.52	0.42–0.64	**<0.001**
Black or Black British	437	83.7	0.48	0.38–0.60	**<.001**	0.57	0.44–0.72	**<0.001**
Chinese	93	86.1	0.57	0.33–0.99	**.05**	0.65	0.37–1.28	0.12
Any other ethnic group	60	81.1	0.40	0.22–0.71	**<.001**	0.48	0.16–0.87	**0.02**
*Ethnic sub-category*								
White British	34,745	91.6	(ref)^d^			(ref)^d^		
White Irish	496	88.6	0.71	0.55–0.92	**.01**	0.76	0.58–0.99	**.04**
Any other White background	712	90.6	0.88	0.69–1.12	.30	0.98	0.77–1.26	.90
White & Black Caribbean	45	81.8	0.41	0.21–0.82	**.01**	0.48	0.24–0.97	**.04**
White & Black African	19	86.4	0.58	0.17–1.96	.38	0.63	0.18–2.16	.46
White & Asian	57	87.7	0.65	0.31–1.37	.26	0.78	0.37–1.66	.52
Any other Mixed background	52	88.1	0.68	0.31–1.50	.34	0.80	0.36–1.77	.58
Indian	304	82.6	0.44	0.33–0.57	**<.001**	0.48	0.36–0.64	**<.001**
Pakistani	121	83.5	0.46	0.30–0.72	**.001**	0.53	0.34–0.82	**.01**
Bangladeshi	23	69.7	0.21	0.10–0.44	**<.001**	0.27	0.13–0.58	**.001**
Any other Asian background	138	87.3	0.63	0.40–1.01	.06	0.75	0.46–1.20	.23
Caribbean	229	82.4	0.43	0.31–0.58	**<.001**	0.50	0.36–0.68	**<.001**
African	184	84.4	0.50	0.34–0.72	**<.001**	0.61	0.42–0.89	**.01**
Any other Black background	24	92.3	1.01	0.26–4.65	.90	1.32	0.31–5.65	.71
Chinese	93	86.1	0.57	0.33–0.99	**.05**	0.65	0.37–1.28	.12
Any other ethnic group	60	81.1	0.40	0.22–0.71	**<.001**	0.48	0.16–0.87	**.02**
(b) Received an understandable answer from a doctor^e^								
*Broad ethnic category*								
White	27,962	83.5	(ref)^c^		(ref)^c^			
Mixed	135	75.0	0.60	0.42–0.83	**.003**	0.64	0.45–0.91	**.01**
Asian or Asian British	427	69.3	0.45	0.38–0.53	**<.001**	0.49	0.41–0.59	**<.001**
Black or Black British	312	75.9	0.63	0.50–0.79	**<.001**	0.72	0.57–0.91	**.01**
Chinese	68	70.8	0.48	0.31–0.75	**.001**	0.51	0.32–0.81	**.004**
Any other ethnic group	55	84.6	1.09	0.56–2.14	.80	1.43	0.72–2.85	.31
*Ethnic sub-category*								
White British	27,007	83.5	(ref)^d^			(ref)^d^		
White Irish	407	84.3	1.06	0.83–1.36	.65	1.11	0.86–1.42	.43
Any other White background	548	80.5	0.81	0.67–0.99	**.04**	0.88	0.72–1.07	.20
White & Black Caribbean	37	69.8	0.46	0.25–0.82	**.01**	0.49	0.27–0.89	**.02**
White & Black African	16	76.2	0.63	0.23–1.73	.37	0.68	0.24–1.89	.46
White & Asian	49	79.0	0.75	0.40–1.37	.35	0.84	0.45–1.58	.59
Any other Mixed background	33	75.0	0.59	0.30–1.17	.13	0.62	0.31–1.24	.18
Indian	226	71.8	0.50	0.39–0.64	**<.001**	0.53	0.41–0.59	**<.001**
Pakistani	97	68.8	0.44	0.31–0.62	**<.001**	0.49	0.34–0.71	**<.001**
Bangladeshi	11	33.3	0.12	0.05–0.20	**<.001**	0.14	0.07–0.30	**<.001**
Any other Asian background	93	73.2	0.54	0.36–0.80	**.002**	0.58	0.40–0.88	**.01**
Caribbean	165	73.3	0.54	0.40–0.73	**<.001**	0.64	0.47–0.87	**.004**
African	132	78.6	0.73	0.50–1.05	.09	0.81	0.55–1.18	.27
Any other Black background	15	83.3	0.99	0.29–3.41	.99	1.28	0.36–4.54	.70
Chinese	68	70.8	0.48	0.31–0.75	**.001**	0.51	0.32–0.81	**.004**
Any other ethnic group	55	84.6	1.09	0.56–2.14	.80	1.43	0.72–2.85	.31
(c) Received an understandable answer from a ward nurse^f^								
*Broad ethnic category*								
White	24,144	75.9	(ref)^c^			(ref)^c^		
Mixed	129	73.7	0.89	0.64–1.25	.50	0.99	0.70–1.40	.964
Asian or Asian British	370	61.9	0.52	0.44–0.61	**<.001**	0.60	0.50–0.71	**<.001**
Black or Black British	262	65.7	0.61	0.49–0.75	**<.001**	0.73	0.59–0.91	**.01**
Chinese	60	59.4	0.46	0.31–0.69	**<.001**	0.53	0.35–0.79	**.002**
Any other ethnic group	46	71.9	0.81	0.47–1.40	.45	1.14	0.65–2.00	.64
*Ethnic sub-category*								
White British	23,319	76.0	(ref)^d^			(ref)^d^		
White Irish	343	75.4	0.97	0.78–1.20	.77	1.04	0.83–1.29	.76
Any other White background	482	73.7	0.87	0.74–1.06	.18	1.01	0.84–1.21	.91
White & Black Caribbean	37	71.2	0.78	0.43–1.42	.42	0.85	0.46–1.56	.60
White & Black African	13	72.2	0.82	0.30–2.31	.71	0.85	0.30–2.44	.77
White & Asian	48	80.0	1.27	0.67–2.38	.47	1.48	0.77–1.51	.23
Any other Mixed background	31	68.9	0.70	0.37–1.32	.27	0.79	0.41–1.51	.48
Indian	194	62.8	0.53	0.42–0.67	**<.001**	0.62	0.48–0.79	**<.001**
Pakistani	83	61.0	0.50	0.35–0.70	**<.001**	0.57	0.40–0.81	**.002**
Bangladeshi	10	34.5	0.16	0.08–0.36	**<.001**	0.24	0.11–0.52	**<.001**
Any other Asian background	83	67.0	0.64	0.44–0.93	**.02**	0.75	0.51–1.10	.14
Caribbean	139	65.9	0.61	0.46–0.81	**.001**	0.76	0.57–1.03	.07
African	111	65.7	0.61	0.44–0.83	**.002**	0.70	0.50–0.97	**.04**
Any other Black background	12	63.2	0.54	0.31–0.69	**<.001**	0.69	0.26–1.79	.44
Chinese	60	59.4	0.46	0.31–0.69	**<.001**	0.53	0.35–0.79	**.002**
Any other ethnic group	46	71.9	0.81	0.47–1.40	.45	1.14	0.65–2.00	.64

Note: Significant associations (*p* < .05) are highlighted in bold.

^a^Adjusted for age, gender, long-standing condition, time since first treatment, tumour group, patient classification, response to treatment, IMD quintile, Trust location (in/outside Greater London) and Trust.

^b^Analysis was restricted to 40,880 respondents and excluded patients who did not respond to Q24 (*n* = 18,931) or had missing data for age, gender, ethnicity, time since first treatment, long-standing condition status, response to treatment or IMD quintile (*n* = 8926). According to the likelihood ratio test, the model using ethnic sub-categories was not significantly better (at 95% confidence level) than the model using broad categories according to the likelihood ratio test (*χ* ^2^(10) = 12.80, *p* = .24).

^c^For comparisons by broad ethnic categories, the reference group was White patients.

^d^For comparisons by ethnic sub-categories, the reference group was White British patients.

^e^Analysis was restricted to 34,877 respondents and excluded patients who did not respond to Q37 (*n* = 26,507) or had missing data for age, gender, ethnicity, time since first treatment, long-standing condition status, response to treatment or IMD quintile (*n* = 7353). According to the likelihood ratio test, the model using ethnic sub-categories was significantly better (at 95% confidence level) than the model using broad categories according to the likelihood ratio test (*χ* ^2^(10) = 18.06, *p* = .05).

^f^Analysis was restricted to 33,138 respondents and excluded patients who did not respond to Q41 (*n* = 28,685) or had missing data for age, gender, ethnicity, time since first treatment, long-standing condition status, response to treatment or IMD quintile (*n* = 6914). According to the likelihood ratio test, the model using ethnic sub-categories was not significantly better (at 95% confidence level) than the model using broad categories according to the likelihood ratio test (χ^2^(10) = 9.74, p = 0.47).

## Discussion

This study investigated the relationship between ethnicity and patients’ reported experience of cancer care with a focus on communication and information provision. Patients’ overall rating of care varied both between and within broad ethnic groups and was correlated with ratings of communication and information provision. There was considerable variation in patients’ reported experiences of communication and information provision across the cancer care pathway and by staff member, as well as by ethnicity. Non-white patients, especially Chinese and Asian (and in particular Bangladeshi) individuals, rated their experiences of communication and information provision less positively than White patients.

Studies have demonstrated that non-White cancer patients are less likely than White patients to rate their overall care and treatment positively (Quality Health 2013; Bone et al. 2014) and the results of our analysis provide additional evidence of such ethnic inequalities. They also go further by highlighting the varied experiences of patients within broad ethnic categories. For example, among Asian patients, those of Bangladeshi ethnicity reported the worst experiences, while, among White patients, those of British or Irish ethnicity were more likely to rate their care positively. There are many hypotheses as to why patients’ ratings of care experiences vary by ethnicity; for example, it has been suggested that ethnic minority patients may receive the same care but rate it more poorly due to different expectations (Lyratzopoulos et al. 2012). However, other studies have presented evidence that expectations of care are in fact similar between ethnic groups, particularly in the area of communication (Weinick et al. 2011). An alternative hypothesis asks if ethnic minority patients have more challenging clinical cases. As this was a secondary analysis of patient experience survey data limited clinical information was available and it was not possible to determine the complexity of patients’ clinical cases, but we were able to control for the effects of tumour group and self-reported response to treatment in our analyses and so it is unlikely that clinical differences could account for the results observed in this study. Other studies have shown that a large proportion of observed variation in care ratings may be due to ethnic minorities being concentrated in poorly performing services (Lyratzopoulos et al. 2012); however, the inclusion of a random effect for NHS Trust of treatment in our multi-level model accounts and adjusts for this potential confounding due to clustering.

Patients from different ethnic groups may also have different preferences for information related to their condition, care and treatment. For example, in a study examining the use of health information among cancer patients in Hawaii, Japanese patients preferred printed materials from healthcare providers, non-Japanese Asian patients preferred person-to-person communication and Caucasian patients preferred online sources (Kakai et al. 2003). There may also be disparities in information preferences between ethnic minority patients and their families. For example, discordance with regards preferences about prognostic information was found among migrant cancer patients and their families in Australia with families preferring non-disclosure (Mitchison et al. 2012). Although these studies were conducted in non-UK settings and involved different ethnic groups, it is likely that similar issues exist amongst minority ethnic groups in England. For example, ethnic minority patients may not be able to access information in their preferred format or receive the information they want, especially if there are language barriers, thereby leading to a less positive experience of care.

Language differences between staff and patients have been suggested as another possible cause of variation in ratings by ethnicity. As the NCPES does not gather information on the languages spoken by respondents, we were not able to investigate or control for the effects of language. According to a study estimating access to NHS translation services (Gill et al. 2009), Bangladeshis have the highest proportion of non-English speakers which suggests language may play a role in cancer patient satisfaction. Yet, this hypothesis does not explain all the patterns observed in our analysis; for example, White Irish patients rated communication with CNSs more positively than White British patients though both groups are native English speakers. Although language can be integral to ethnicity, there are many other social parameters that contribute to ethnic groupings including external aspects, such as communities and networks, and internal aspects, such as a sense of identity and values, group obligations and feelings of security and comfort (Isajiw 1993). Thus, ethnic variation in ratings may be attributable to several factors. Evidence from studies related to maternity care indicate that differences in language may in fact have less influence on care ratings than cultural barriers and staff stereotyping (Puthussery et al. 2010). Different ethnic group practices can be difficult for outsiders to understand; for example, in a study exploring communication with ethnic minorities in primary care, staff found it challenging to understand and accommodate Bangladeshi patients’ needs to fit appointments around particular work requirements including ‘restaurant hours’ (Hawthorne, Rahman, and Pill 2003). However, there is evidence to suggest that healthcare professionals who build up long-term relationships with Bangladeshi patients begin to understand and be more tolerant of their particular needs than their colleagues (Hawthorne, Rahman, and Pill 2003). The finding from our study, that Bangladeshi participants-rated communication with a CNS more positively than with other staff groups, may indicate that a long-term, personal relationship with a named staff member improves communication among ethnic minority individuals. The mode of communication may also be an important factor in explaining the poor communication ratings from Bangladeshi patients observed in this study as Sylheti, spoken by many Bangladeshis, has no agreed written form (Duff, Lamping, and Ahmed 2001) and so individuals may not have access to understandable written information.

The data used in this analysis was taken from a national survey (2012–2013 NCPES) with a large sample size (*N* = 68,737) and a relatively high response rate (64%). Patients’ responses were dichotomised as per the survey guidance thereby facilitating comparison of results with other studies based on this data set, and the data set was large enough to allow analysis by ethnic sub-category to reveal patterns previously masked by the broad categories more commonly used in research. Despite these strengths, there are several limitations to this study. For example, non-White patients are known to be less likely to respond to NCPES, potentially introducing selection bias and, although language support leaflets were available for survey participants, the NCPES was written in English and therefore patients with low English skills are less likely to respond and would be excluded from the study. Also, the phrasing of some questions in the NCPES makes it impossible to unpick the mode of communication or information provision the patient is rating. For example, when responding to Q25 ‘Did hospital staff give you information about support or self-help groups for people with cancer?’ patients may be rating verbal and/or written communication. Finally, as the dataset is cross-sectional there is a possibility of recall bias.

This analysis offers insight into successes and failings in different areas of communication along the cancer care pathway; however further work is needed to understand the causes of these inequalities. Interviews or focus groups with minority ethnic groups living in the UK may be useful to understand further their experiences of care. In particular, to explore the inequalities in patient experience reported by Bangladeshi patients observed in this study, focus groups or interviews in Sylheti may be useful (Duff, Lamping, and Ahmed 2001). Additionally, surveys which record both ethnicity and language of preference may provide insight into the role of language in patient experience.

Regardless of the causes, the inequalities identified in our analysis highlight challenges in communicating with and providing care to a diverse patient population and illustrate the need for services to adapt in order to provide the best possible care to all patients, regardless of ethnicity. These inequalities may also indicate that previously implemented policies, such as national protocols for delivery of information related to treatment and operations, have been insufficient. It is important to note that effective communication is not simply about giving all the relevant information to a patient, but also about tailoring information delivery to the needs, beliefs and values of the individual (Watts et al. 2004). Services should therefore be adapted to improve communication in the presence of language and/or cultural variation and policy-makers need to be aware of the inequalities that exist and ensure that new policies address these issues and support individuals in most need (El Ansari et al. 2009). Alternative methods of information provision, tailored to specific needs and preferences of minority ethnic groups may prove effective, such as audio or video information in Sylheti for Bangladeshi patients who find understanding written information challenging (Thomas et al. 1999). The education of healthcare professionals, from students to consultants, should address ethnic bias (Dedier et al. 1999) and the need to understand patients as individuals (Kai et al. 1999) so as to ensure the best possible care and patient experience.

## Disclosure statement

No potential conflict of interest was reported by the authors.

## Key messages

(1) Cancer patient experience is known to vary by ethnicity, but variation within the broad ethnic categories typically used in research is poorly-described.(2) This secondary analysis of data from the 2012–2013 National Cancer Patient Experience Survey provides evidence of variation in patients’ ratings of communication and information provision – key domains of patient experience – both between and within ethnic categories.(3) Compared to White patients, Non-White patients (particularly Asian patients) were less likely to report positive experiences, and among Asian patients those of Bangladeshi ethnicity reported the poorest experiences.(4) Further work to understand the causes of variation in cancer patients' experiences of information provision and communication is required to address ethnic inequalities at practice and policy level.

## APPENDIX

**Table A1:** 2012–2013 NCPES questions related to communication and information provision with details of the dichotomised responses.

			Positive		Negative		
	*Diagnosis*						
Stage of cancer care pathway	13	Did you understand the explanation of what was wrong with you?	Yes, I completely understood it		Yes, I understood some of it	No, I did not understand it	
	14	When you were told you had cancer, were you given written information about the type of cancer you had?	Yes, and it was easy to understand		Yes, but it was difficult to understand	No, I was not given written information about the type of cancer I had	
	*Diagnostic tests*						
	6	Beforehand, did a member of staff explain the purpose of the test?	Yes, completely		Yes, to some extent	No, but I would have liked an explanation	
	7	Beforehand, did a member of staff explain what would be done during the test procedure?	Yes, completely		Yes, to some extent	No, but I would have liked an explanation	
	8	Beforehand, were you given written information about your test?	Yes, and it was easy to understand		Yes, but it was difficult to understand	No, but I would have liked written information about the test(s)	
	9	Were the results of the test(s) explained in a way you could understand?	Yes, completely		Yes, to some extent	No, but I would have liked an explanation	
	*Treatment side effects*						
	17	Were the possible side effects of treatment(s) explained in a way you could understand?	Yes, definitely		Yes, to some extent	No, side effects were not explained	
	18	Before you started your treatment, were you given written information about the side effects of treatment(s)?	Yes, and it was easy to understand		Yes, but it was difficult to understand	No, I was not given written information about side effects	
	19	Were you also told about any side effects of the treatment that could affect you in the future rather than straight away?	Yes definitely		Yes, to some extent	No, future side effects were not explained	
	*Operations*						
	33	Before you had your operation, did a member of staff explain what would be done during the operation?	Yes, completely		Yes, to some extent	No, but I would have liked an explanation	
	34	Beforehand, were you given written information about your operation?	Yes, and it was easy to understand		Yes, but it was difficult to understand	No, but I would have liked an explanation	
	35	After the operation, did a member of staff explain how it had gone in a way you could understand?	Yes, completely		Yes, to some extent	No, but I would have liked an explanation	
	*Support*						
	25	Did hospital staff give you information about support or self-help groups for people with cancer?	Yes		No, but I would have liked information		
	26	Did hospital staff discuss with you or give you information about the impact cancer could have on your work life or education?	Yes		No, but I would have liked a discussion or information		
	27	Did hospital staff give you information about how to get financial help or any benefits you may be entitled to?	Yes		No, but I would have liked information		
	28	Did hospital staff tell you that you could get free prescriptions?	Yes		No, but I would have liked information		
	*Leaving hospital*						
	53	Were you given clear written information about what you should or should not do after leaving hospital?	Yes		No		
	54	Did hospital staff tell you who to contact if you were worried about your condition or treatment after you left hospital?	Yes		No		
General	*Verbal communication with staff*						
	24	When you have important questions to ask your Clinical Nurse Specialist, how often do you get answers you can understand?	All or most of the time		Some of the time	Rarely or never	
	37	When you had important questions to ask a doctor, how often did you get answers that you could understand?	All or most of the time		Some of the time	Rarely or never	
	41	When you had important questions to ask a ward nurse, how often did you get answers you could understand?	All or most of the time		Some of the time	Rarely or never	
	*Overall*						
	67	How much information were you given about your condition and treatment?	The right amount		Not enough	Too much	
	70	Overall, how would you rate your NHS care?	Excellent	Very good	Good	Fair	Poor
